# A238 BEST APPROACH TO SURVEILLANCE COLONOSCOPY FOR COLORECTAL CANCER IN PATIENTS WITH PRIMARY SCLEROSING CHOLANGITIS AND INFLAMMATORY BOWEL DISEASE

**DOI:** 10.1093/jcag/gwad061.238

**Published:** 2024-02-14

**Authors:** R Khan, G Dhalwi, A Ricciuto

**Affiliations:** The Hospital for Sick Children Department of Paediatrics, Toronto, ON, Canada; The Hospital for Sick Children Department of Paediatrics, Toronto, ON, Canada; The Hospital for Sick Children Department of Paediatrics, Toronto, ON, Canada

## Abstract

**Background:**

Primary sclerosing cholangitis (PSC) is a chronic, cholestatic liver disease and it has been recognized as a risk factor for hepatobiliary and colorectal carcinoma (CRC). Inflammatory bowel disease (IBD) is associated with a 6-fold increased risk of CRC as compared to the general population but with co-existing PSC, this risk rises to 30-fold and hence CRC surveillance is critical in these patients.

**Aims:**

We performed a systematic review of the literature on surveillance colonoscopy for CRC in patients with PSC-IBD, with the aim of identifying the most effective approach.

**Methods:**

The protocol was registered in the International Prospective Register of Systematic Reviews (PROSPERO) under ID 472215. The search was executed in the databases Medline, EMBASE, Cochrane, Web of science, Scopus and Google Scholar databases from 1947 to May 2023. Observational or randomized controlled studies were included if patients had PSC-IBD (paediatric or adult) undergoing surveillance colonoscopy. There was no restriction for language. We used a modified version of the Newcastle–Ottawa Quality Assessment Scale to assess the risk of bias. Neoplasia was defined as the presence of low-grade dysplasia, high grade dysplasia or colorectal carcinoma.

**Results:**

Amongst 2900 studies identified, 7 studies met the inclusion criteria with a total number of 379 PSC-IBD patients and 1884 IBD patients (all adult). The median frequency of surveillance colonoscopy was every 0-2 years. The most common endoscopic presentation of dysplasia in PSC-IBD was a normal appearing mucosa or subtle abnormalities such as mild inflammation. Three studies performed random and targeted biopsies and only one study did quadruple random biopsy (every 10 cm of the colon). Two studies used chromoendoscopy and high-definition endoscopy whereas another two studies used white light colonoscopy. Studies varied in terms of the number of biopsies taken and timing for the frequency and initiation of surveillance. Outcomes included intensive surveillance, resection, and colectomy. Our meta-analysis of 2 studies that reported the prevalence of colorectal dysplasia in PSC-IBD patients revealed a pooled prevalence of 21% (95% CI 1.4%-28%, I^2^ = 0%, Pampersand:003C0.597, shown in Figure 1)

**Conclusions:**

Colorectal dysplasia is prevalent in patients with PSC-IBD and is associated with worse disease outcomes. As a result, PSC-IBD patients should be screened annually from the diagnosis of PSC. However, there is variation in practice, and no clear consensus on the best approach to perform screening colonoscopy in this population. Literature comparing various approaches is very sparse. Additional high-quality studies on this subject are needed to guide practice, particularly in paediatrics where data are non-existent.

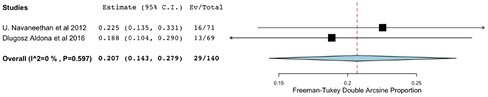

Figure 1: Forest plot for meta-analysis of CRC dysplasia prevalence in PSC-IBD

**Funding Agencies:**

None

